# A learning-based image processing approach for pulse wave velocity estimation using spectrogram from peripheral pulse wave signals: An *in silico* study

**DOI:** 10.3389/fphys.2023.1100570

**Published:** 2023-03-03

**Authors:** Juan M. Vargas, Mohamed A. Bahloul, Taous-Meriem Laleg-Kirati

**Affiliations:** ^1^ Computer, Electrical, and Mathematical Sciences and Engineering, King Abdullah University of Science and Technology (KAUST), Makkah, Saudi Arabia; ^2^ Electrical Engineering Department, Alfaisal University, Riyadh, Saudi Arabia; ^3^ National Institute for Research in Digital Science and Technology INRIA, Saclay, France

**Keywords:** pulse wave velocity, spectrogram, PPG, distal blood pressure, machine learning (ML), image processing, semi-classical signal analysis

## Abstract

Carotid-to-femoral pulse wave velocity (cf-PWV) is considered a critical index to evaluate arterial stiffness. For this reason, estimating Carotid-to-femoral pulse wave velocity (cf-PWV) is essential for diagnosing and analyzing different cardiovascular diseases. Despite its broader adoption in the clinical routine, the measurement process of carotid-to-femoral pulse wave velocity is considered a demanding task for clinicians and patients making it prone to inaccuracies and errors in the estimation. A smart non-invasive, and peripheral measurement of carotid-to-femoral pulse wave velocity could overcome the challenges of the classical assessment process and improve the quality of patient care. This paper proposes a novel methodology for the carotid-to-femoral pulse wave velocity estimation based on the use of the spectrogram representation from single non-invasive peripheral pulse wave signals [photoplethysmography (PPG) or blood pressure (BP)]. This methodology was tested using three feature extraction methods based on the semi-classical signal analysis (SCSA) method, the Law’s mask for texture energy extraction, and the central statistical moments. Finally, each feature method was fed into different machine learning models for the carotid-to-femoral pulse wave velocity estimation. The proposed methodology obtained an $R^2^\geq0.90$ for all the peripheral signals for the noise-free case using the MLP model, and for the different noise levels added to the original signal, the SCSA-based features with the MLP model presented an $R^2^\geq0.91$ for all the peripheral signals at the level of noise. These results provide evidence of the capacity of spectrogram representation for efficiently assessing the carotid-to-femoral pulse wave velocity estimation using different feature methods. Future work will be done toward testing the proposed methodology for *in-vivo* signals.

## 1 Introduction

Carotid-to-femoral pulse wave velocity (cf-PWV) is considered a critical index to evaluate arterial stiffness. For this reason, estimating Carotid-to-femoral pulse wave velocity (cf-PWV) is essential for diagnosing and analyzing different cardiovascular diseases. Despite its broader adoption in the clinical routine, the measurement process of cf-PWV is considered a demanding task for clinicians and patients making it prone to inaccuracies and errors in the estimation. A smart non-invasive, and peripheral measurement of cf-PWV could overcome the challenges of the classical assessment process and improve the quality of patient care. This paper proposes a novel methodology for the cf-PWV estimation based on the use of the spectrogram representation from single non-invasive peripheral pulse wave signals [photoplethysmography (PPG) or blood pressure (BP)]. This methodology was tested using three feature extraction methods based on the semi-classical signal analysis (SCSA) method, the Law’s mask for texture energy extraction, and the central statistical moments. Finally, each feature method was fed into different machine learning models for the cf-PWV estimation. The results obtained for each feature method provide evidence of the capacity of spectrogram representation combined with machine learning models as an intelligent tool for efficiently assessing the cf-PWV estimation. Cardiovascular diseases (CVDs) are the leading cause of mortality worldwide, with 17.9 million deaths in 2019, representing 32% of all global deaths, (Mensah et al., 2019). Patients at risk of evolving CVDs are assessed by evaluating different bio-markers ranging from age and sex to arterial stiffness (AS), (Gade et al., 2021). Arterial stiffness is considered one of the highest risk markers and has attracted much attention in clinical and experimental studies, (van Sloten et al., 2014). Arterial stiffness depicts the rigidity of the arterial vessels, positively associated with arterial pulse pressure, which can significantly affect the heart and vascular physiology.

Over the last decades, a myriad of techniques for evaluating AS have been explored and validated, some of which are more widely functional nowadays in clinical practice than others, for example, the cardio-ankle vascular index that reflects the stiffness from the ascending aorta to the ankle arteries (Matsushita et al., 2019), the pulse pressure defined as the difference between the diastolic and systolic pressure (Mackenzie et al., 2002) assessing arterial stiffness. It is usually evaluated by dividing the distance traveled by the pulse wave between two arterial sites divided by the time taken to travel the distance (path length between the two sites). When the two arterial sites are the carotid and femoral sites, then we refer to the Carotid to femoral pulse wave velocity (cf-PWV) and usually provide information on the central arterial stiffness. The feasibility of cf-PWV in evaluating vascular stiffness has been validated through a strong correlation with major parameters and conditions such as hypertension severity levels, vascular aging, and atherosclerosis (Blacher et al., 1999; Shokawa et al., 2005; Mattace-Raso et al., 2006; Willum Hansen et al., 2006; Choi et al., 2007; Kim and Kim, 2019). Despite the crucial role of cf-PWV, there is no reliable method for estimating the cf-PWV. Most methods that exist in the literature have limitations. For instance, they rely on experienced personnel to realize the correct measurement, consisting in acquiring the carotid and femoral pressure waveform and measuring the traveling distance, as highlighted in (Matsushita et al., 2019). The measurement can therefore be subject to errors and inaccuracies, in particular, when evaluating the path length between the carotid and femoral sites (Tavallali et al., 2018). Further discussions on the measurement modalities and the main advantages and limitations of cf-PWV measurements can be read in (Rajzer et al., 2008).

Recent papers have investigated the use of Artificial Intelligence (AI) in estimating pulse wave velocity where non-invasive available measurements are used. AI-based approaches present the advantage of estimating the cf-PWV from non-invasive measurements which can be incorporated into the clinical routine without involving any complex protocol or experienced personnel. For instance, a non-calibrated carotid tonometry pressure waveform has been combined with a clinical routine variable to feed a machine learning model with *Intrinsic Frequency* features (Tavallali et al., 2015). Recently, a multi-layers perceptron-based cf-PWV estimation using fiducial points-based features extracted from the photoplethysmogram (PPG) signal and its first, second, and third derivatives, has been proposed. Another investigation by Weiwei et al., in (Jin et al., 2021), has proposed two machine learning pipelines, namely the *Gaussian process regression* and *Recurrent Neural Network* for the cf-PWV estimation from the radial blood pressure waveform. The two proposed machine learning pipelines used key features generated from the timing and magnitude of the fiducial points and the heart rate. More recently, in 2022, (Garcia et al., 2022) used a Multiple Linear Regression model to study the feasibility of the Semi-Classical Signal Analysis (SCSA)-based features extracted from Blood Pressure (BP) and PPG signals extracted from peripheral locations. In this study, feature extraction from a two-dimensional signal representation of the BP and PPG signals improved the estimation accuracy and robustness compared to the original one-dimensional signals’ results. Finally, (Li et al., 2022) proposed the cf-PWV prediction based on the XGBoost algorithm using wrist photoplethysmogram (wPPG) signals acquired from wearable devices. Despite the promising results, AI-based algorithms are not yet reliable and require improvements in terms of accuracy but also in terms of the universality of the algorithms, which refers to the fact that they can perform well for data that have not been considered in the training of the machine learning model.

In this paper, our objective is to contribute to improving AI-based algorithms for the estimation of the cf-PWV by proposing the use of spectrograms of pulse wave signals instead of one-dimensional signals. We believe that using the spectrogram, which provides both temporal and frequency dimensions of the signal, will help in improving the accuracy of measuring cf-PWV and therefore would help in including the cf-PWV measure in the clinical routine practice without the need for an expert. The use of spectrogram representation on PPG signals has been studied in the past proving good performance over different applications. In 2020, (Donida Labati et al., 2021) used a *SVM* model with features extracted from the PPG spectrogram for biometric recognition. Another use of PPG spectrogram representation is presented by (Siam et al., 2021) where they use the spectrogram as an input image for Blood Pressure estimation using *Siamese networks* and *Convolutional neural networks* (CNN).

**FIGURE 1:**
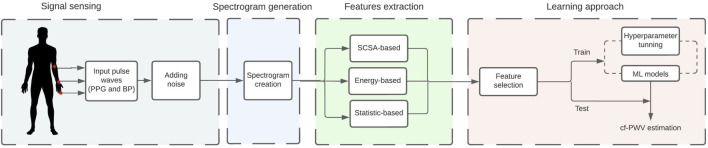
Schematic illustration of the estimation pipeline of the carotid-to-femoral pulse wave velocity based on pulse wave images. PPG indicates the photoplethysmography signals, BP the blood pressure signals and ML indicates machine learning models.

This work used the spectrogram representation from peripheral signals for cf-PWV estimation using three feature methods. The first feature type was based on the Semi-Classical Signal Analysis (SCSA) method that relies on the Schrodinger operator’s spectral problem. The second type was based on the Law’s mask filters that compute the energy texture of an image, and the third was based on the central moments that give a statistical description of the image. Finally, these features were fed individually to different machine learning models to obtain the final estimation. Figure 1 illustrates the proposed estimation pipeline of the cf-PWV.

## 2 Materials and methods

### 2.1 Materials

#### 2.1.1 Dataset

Due to the absence of real hemodynamic data to validate and test the proposed approach, in this paper, we used an *in silico* hemodynamic public database^1^. A pre-validated one-dimensional model has been used for generating the database of simulated pulse wave signals at different arterial locations. This model generates the signals based on different cardiovascular properties such as age, heart rate, blood density, and arterial diameter among other cardiovascular parameters (Charlton et al., 2019). These signals are often used to evaluate various pre-clinical assessment studies and hemodynamic analyzer algorithms, such as assessing pulse wave velocity. The database emulates pulse wave signals of one cardiac cycle of length from 4,374 virtual healthy adults with different ages between 25 and 75 in 10-year increments (six age groups) and heart rate between 66 bpm and 86 bpm, using a sampling frequency of 500 Hz for each signal. Each group has 729 virtual subjects’ pulse waves with distinct cardiac and arterial parameters like arterial stiffness and heart rate within normal ranges. In this study, PPG and BP waveforms at the level of the brachial, radial, and digital arteries were used to create the spectrograms and estimate the cf-PWV.

### 2.2 Method

The proposed methodology is summarized in Figure 1. A spectrogram is created using pulse wave signals from the *in silico* data. Then, features are extracted from three different methods and are fed individually into the learning approach stage. The best features were selected to train the model and estimate the cf-PWV value.

#### 2.2.1 Spectrogram generation

The spectrogram is a time-frequency representation used to analyze the change of frequency with respect to time from a given input signal. In this paper, spectrograms are generated using the function *spectrogram* from MATLAB. It is well known that the selection of parameters involved in the creation of the spectrogram will define the quality of the representation, as shown in (Jablonski and Dziedziech, 2022). For this reason, windows’ parameters, spectrogram’s shape, and overlapping percentage were finely tuned.

Regarding the window type, it has been shown that Hamming and Kaiser’s windows are good options for generating spectrograms from pulse wave signals such as PPG (Zong and Jafari, 2015; Esgalhado et al., 2021). Overlapping percentage values of 0, 60, and 95 for Hamming windows and 0, 61, and 70 for Kaiser, were selected based on the values reported in (Trethewey, 2000) and (Heinzel et al., 2002). However, for the Kaiser window, it is necessary to define an extra parameter called *α* which changes depending on the overlapping percentage (Heinzel et al., 2002). Values of 0.5, 3, and 5 were used for the 0, 61, and 70 overlapping percentages, respectively. Additionally, the shape selection was made, considering the requirement from the 2D-SCSA feature extraction method to use square images (Kaisserli and Laleg-Kirati, 2014). For this reason, a squared spectrogram of sizes 250, 166, 100, 50, and 20 was used.

Finally, to select the final values, the spectrogram quality coefficients used in (Jablonski and Dziedziech, 2022) were calculated for each combination of parameters, as follows,

$$Q_{f}=\frac{1}{F}\overset{F}{\underset{f=1}{\sum }}\frac{\sigma_{f}\left[f\right]}{\mu_{f}\left[f\right]}\;\;\;Q_{t}=\frac{1}{T}\overset{T}{\underset{t=1}{\sum }}\frac{\sigma_{t}\left[t\right]}{\mu_{t}\left[t\right]}\;\;\;Q_{tf}=Q_{f}Qt$$ (1)

where *t* and *f* represent the time (rows) and frequency (columns) of the spectrogram, and *T* and *F* are the number of time and frequency points respectively. *σ* represents the standard derivation, *μ* is the mean. Higher values in these metrics represent a better capacity of the spectrogram to represent the variability of a given signal.

Finally, the combination of parameters with the higher values for the metrics was obtained using the Hamming window, with 0% of overlapping and an image size of 250 × 250 pixels. More details on the parameters tuning and obtained results for all the combinations can be found in the Supplementary Material.

#### 2.2.2 Semi-classical signal analysis method

##### 2.2.2.1 Definition

The semi-classical signal analysis (SCSA) method has been proposed in (Laleg-Kirati et al., 2013) for pulse-shaped signal reconstruction, denoising, and characterization, where the signal is decomposed into a set of signal-dependent adaptive squared eigenfunctions of the Schrödinger operator. The SCSA method has been successfully used for features extraction of blood pressure (BP) and PPG signals by (Laleg-Kirati et al., 2013; Li and Laleg-Kirati, 2021a; Garcia et al., 2022), showing the feasibility of this method to provide useful information on the shape of the input signal which helps to detect morphology changes in the signal. This method has been extended to image representation (Kaisserli and Laleg-Kirati, 2014), denoising (Chahid et al., 2017; Chahid et al., 2018), and feature extraction (Garcia et al., 2022).

Definition 1 Let I (*x, y*) be a positive real valued square matrix, the image representation I_2h_ of I (*x, y*) using the 2D-SCSA is defined as follows*:*

$$I_{2h}\left(x,y\right)={\left[\frac{h^{2}}{L_{2,\gamma }^{cl}}\overset{M_{h}}{\underset{m=1}{\sum }}{\left(-\lambda_{mh}\right)}^{\gamma }\Psi_{mh}^{2}\left(x,y\right)\right]}^{\frac{1}{\gamma +1}}$$ (2)

where $h\;\in R_{+}^{*}$ is known as the semi-classical signal parameter, $\gamma \in R_{+}$ .*λ*_*mh*_ are the negative eigenvalues, and $\Psi_{1h},\Psi_{2h},\ldots ,\Psi_{M_{h}}$ correspond to their associated *L* ^2^-normalized eigenfunctions (*m* = 1, *…*, *M*_*h*_ the number of eigenvalues) extracted from the two-dimensional semi-classical Schrodinger operator described as follows:

$$H_{2,h}\left(I_{2}\right)\psi =-h^{2}\left(\frac{\partial^{2}\psi }{\partial x^{2}}+\frac{\partial^{2}\psi }{\partial y^{2}}\right)-I\psi$$ (3)

and $L_{2,\gamma }^{cl}$ is the suitable semi-classical constant defined as:

$$L_{2,\gamma }^{cl}=\frac{1}{{\left(2\sqrt{\pi }\right)}^{2}}\frac{\Gamma \left(\gamma +1\right)}{\Gamma \left(\gamma +2\right)}$$ (4)

where Γ is the Gamma function.

##### 2.2.2.2 Numerical computation

The 2D-SCSA requires the computation of eigenvalues and eigenfunctions from a 2D operator, leading to a complex and time-consuming process. To reduce the computational burden, a separation of variables approach has been proposed in (Kaisserli and Laleg-Kirati, 2014) where the standard 1D-SCSA is used for each row and each column; the results are then combined using the following formula: (Kaisserli and Laleg-Kirati, 2014):

$$\begin{array}{ll} I_{h,\gamma }\left[i,j\right] & ={\left[\frac{h^{2}}{L_{2,\gamma }^{cl}}\overset{K_{h}}{\underset{k=1}{\sum }}\;\overset{R_{h}}{\underset{r=1}{\sum }}{\left(-\left(\beta_{i,k,h}+\rho_{j,r,h}\right)\right)}^{\gamma },\times \psi_{i,k,h}^{2}\left[j\right]\phi_{j,r,h}^{2}\left[i\right]\right]}^{\frac{1}{1+\gamma }} \end{array}$$ (5)

where *β*_*i*,*k*,*h*_, *k* = 1, …, *K*_*h*_ and *ρ*_*j*,*m*,*h*_, *m* = 1, …, *M*_*h*_ are the eigenvalues for each row and each column respectively, with $\psi_{kh}^{2}\left[j\right]$ and $\phi_{mh}^{2}\left[i\right]$ are the corresponding eigenvectors.

##### 2.2.2.3 Parameters selection

The semi-classical parameter *h* and the parameter *γ* play crucial roles in the SCSA representation as described in (Laleg-Kirati et al., 2013; Kaisserli and Laleg-Kirati, 2014). When the *h* value tends to 0, the SCSA reconstruction converges to the original image producing the best result. However, it has been noticed that the number of eigenvalues is restricted by the number of samples (Piliouras, 2020). Even knowing that the SCSA representation improves when *h* tends to 0, this value cannot be very small as it also depends on the number of samples. In addition, selecting the parameter *γ* given a specific *h* is also important since it affects the intensity values of the reconstructed images. It has been found that for small *h* values, *γ* tends to increase; in contrast, when *h* increases, *γ* tends to decrease.

An appropriate *h* interval has been proposed in (Piliouras, 2020) for 1D-signals, where a minimum value for *h* based on the sampling theorem is introduced. This value had been successfully used in (Piliouras, 2020; Li et al., 2021), providing good accuracy for signal representation. This minimum value is defined as:

$$h_{\min}=\frac{T_{s}}{\pi }\sqrt{V_{\max}}$$ (6)

where *V*_max_ is the maximum value of the input signal and *T*_*s*_ is the sampling period of the images.

**FIGURE 2:**
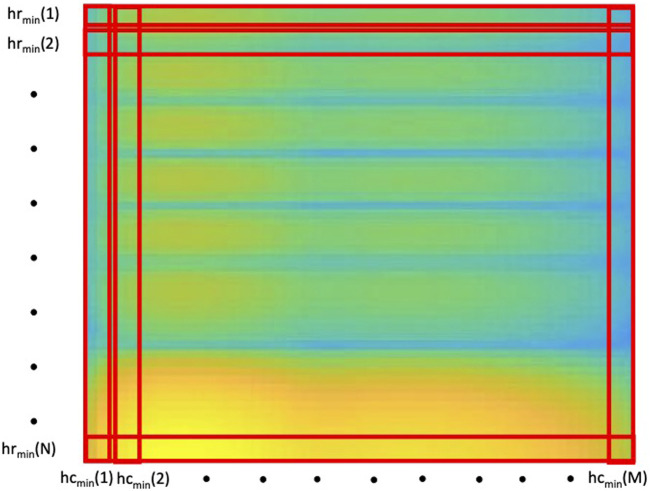
Computation of the 2D-SCSA’s design parameters *h*. *hr*_min_ and *hc*_min_ represent the *h*_min_ values extracted from every row and every column of the spectrogram respectively.

In this paper, we propose to extend the idea of using the *h*_min_ to image representation. *h*_min_ is computed for all the rows (*hr*_min_) and columns (*hc*_min_) in the image, as is shown in Figure 2. However, given that each pixel is affected by the *h* value taken from columns and rows, the mean between these two values was computed to obtain the *h* value (*hm*_min_) for each pixel in the image.

$$hm_{\min}\left(i,j\right)=\frac{hr_{\min}\left(i\right)+hc_{\min}\left(j\right)}{2}\;i=1..N\;j=1..M$$ (7)

To obtain a single *h* value for the entire image $\left({\hat{h}}_{\min}\right)$ , the maximum value from *hm*_min_ matrix was selected to avoid a bad representation or aliasing in the representation.

$${\hat{h}}_{\min}=\max\left(hm_{\min}\right)$$ (8)

The value of the *γ* parameter was selected by a sensitivity analysis. Different values of *γ* have been tested to maximize the structural similarity index measure (SSIM) and the Peak Signal to Noise ratio (PSNR) between the original spectrogram and the reconstructed one using 2D-SCSA. More information about the obtained results from the sensitivity analysis is presented in the Supplementary Material.

#### 2.2.3 Features extraction

In this study, three different feature methods were computed. Obtaining 36 SCSA-based features, 102 Energy-based features, and 6 Statistic based features for the PPG and BP spectrograms for the Radial, Digital, and Brachial locations. Each feature was fed separately into the feature selection method to finally be combined with the different machine learning algorithms to estimate the cf-PWV values. The features used in this study are shown in Table 1, and with the relevant advantages and disadvantages of the three types of features.

**TABLE 1:** Features computed by each type.

Type	Features	Description	Advantages/limitations
SCSA	*INV*_1_, *INV*_2_, *INV*_3_	Three first invariants consisting of some momentum	SCSA has shown great performance as a feature extraction method of pulse wave signals in different applications Laleg-Kirati et al. (2013), Li and Laleg-Kirati (2021a), Garcia et al. (2022). ṠCSA can filter the signal at the same time that extracts the features.
		of the negative eigenvalues Laleg-Kirati et al. (2013).	
			This makes these features robust against noisy signals Li and Laleg-Kirati (2021b), Li et al. (2021), Garcia et al. (2022). Ṫhe limitation of this feature extraction method is the computational complexity causing high running times.
	*K*_*n*_	Mean value of the three first kappas Laleg-Kirati et al. (2013), Li and Laleg-Kirati (2021a).	
	*E*_*n*_	Mean of the three first eigenvalues Laleg-Kirati et al. (2013), Li and Laleg-Kirati. (2021b).	
	*R*_*h*_	Ratio between the first *κ* of the eigenvalues matrix and *h* Li et al. (2021).	
	*M*_*h*_	Ratio between the median of the *κ* of all eigenvalues and *h* Li et al. (2021).	
	*N*_*h*_	Number of eigenvalues Garcia et al. (2022)	
	Mean(*κ*)	Mean value of the kappas Garcia et al. (2022).	
	STD(*κ*)	Stand deviation from Kappas Garcia et al. (2022).	
Energy	*ME* (*E*_*mask*_)	Mean value of the *E*_*mask*_ extracted for the 3 × 3 masks	Law’s mask has shown great performance as a texture feature extraction method for biomedical images and spectrograms Rachidi et al. (2008), Wang (2014), Dash and Jena (2017)
		and 5 × 5 masks Rachidi et al. (2008).	L̇aw’s mask has shown robustness against noise and morphological changes in the images Rachidi et al (2008), Wang (2014), Dash and Jena (2017).
	*STD* (*E*_*mask*_)	Standard deviation of the *E*_*mask*_ extracted for the 3 × 3 masks and 5 × 5 masks Rachidi et al. (2008).	
	*EN* (*E*_*mask*_)	Entropy of the *E*_*mask*_ extracted for the 3 × 3 masks and 5 × 5 masks Rachidi et al. (2008).	
Stratistic	F1	Log base 10 of the standard deviation of the spectrogram Mulimani and Koolagudi (2018).	Statistical features based on central moments have been used as features extraction for event classification and inference detection using spectrograms Dennis et al. (2011), Mulimani and Koolagudi (2018), Oh and Kim (2019)
			Ṡtatistical features are highly interpretable and easy to implement.
	F2	Skewness of the spectrogram Mulimani and Koolagudi (2018).	Ṫhe main limitation is the robustness against noisy data.
	F3	Log base 10 of the kurtosis of the spectrogram Mulimani and Koolagudi (2018).	
	F4	Standar deviation of the normalize spectrogram (Mulimani and Koolagudi, 2018).	
	F5	Skewness of the normalize spectrogram Mulimani and Koolagudi (2018).	
	F6	Kurtosis of the normalize spectrogram Mulimani and Koolagudi (2018).	

##### 2.2.3.1 SCSA-based features

2D-SCSA features were considered to be the eigenvalues computed from each of the three following eigenvalues matrices obtained after applying the 2D-SCSA on the spectrogram:

1. Matrix composed by the extracted eigenvalues from the rows of the spectrogram.
2. Matrix composed of the extracted eigenvalues from the columns of the spectrogram.
3. Matrix composed by the sum of the matrices above.

The first features calculated were the three first invariants proposed by (Laleg-Kirati et al., 2013), consisting of some momentum of the negative eigenvalues. In this work, we compute the invariants for all three cases above: row eigenvalues, column eigenvalues, and combined eigenvalues:

$$\begin{array}{cc} INV_{1} & =4{\hat{h}}_{\min}\overset{N_{h}}{\underset{n=1}{\sum }}\overset{M_{h}}{\underset{m=1}{\sum }}\kappa_{h}\left[m,n\right],\;\;\\ INV_{2} & =\frac{16{\hat{h}}_{\min}}{3}\overset{N_{h}}{\underset{n=1}{\sum }}\overset{M_{h}}{\underset{m=1}{\sum }}\kappa_{h}{\left[m,n\right]}^{3}, \end{array}$$ (9)

$$INV_{3}=\frac{256{\hat{h}}_{\min}}{7}\overset{N_{h}}{\underset{n=1}{\sum }}\overset{M_{h}}{\underset{m=1}{\sum }}\kappa_{h}{\left[m,n\right]}^{7}$$ (10)

where ${\hat{h}}_{\min}$ is the semi-classical constant used for the reconstruction as described in the previous section. *N*_*h*_ is the number of eigenvalues (or columns) and *M*_*h*_ represents the number of rows in the previous eigenvalue matrices. For each matrix, we denote appropriate *κm*, *n*, *h* values depending on the corresponding eigenvalue problem (row, column, or combined) as follows:

$$\kappa {\left[m,n\right]}_{h}={\left(-\lambda_{mh}\right)}^{\gamma }$$ (11)

These invariant parameters were used by (Laleg-Kirati et al., 2010; Li and Laleg-Kirati, 2021b; Garcia et al., 2022) for signal processing features extraction and in (Garcia et al., 2022) for image processing features extraction. This shows the feasibility of these invariants to obtain relevant information from pulse wave signals such as BP and PPG.

Furthermore, based on the first three eigenvalues that approximate the general profile of the image (Laleg-Kirati et al., 2013; Li and Laleg-Kirati, 2021a), the mean value of the three first eigenvalues and *κ* for each matrix were calculated as follows:

$$K_{n}=\frac{1}{M_{h}}\overset{M_{h}}{\underset{m=1}{\sum }}\kappa_{h}\left[i,n\right],\;\;\;\;E_{n}=\frac{1}{M_{h}}\overset{M_{h}}{\underset{m=1}{\sum }}{\left(\kappa_{h}\left[i,n\right]\right)}^{\frac{1}{\gamma }}\;\;\;\;n=\left[1,2,3\right]$$ (12)

We also considered other features as described in (Li et al., 2021) and which consists of the ratio between the first *κ* of the eigenvalues matrix and ${\hat{h}}_{\min}$ (*R*_*h*_) and the ratio between the median of the *κ* of all eigenvalues (*MR*_*h*_), as following:

$$R_{h}=\frac{\kappa_{1h}}{{\hat{h}}_{\min}},\;\;\;MR_{h}=\frac{median\left(\kappa_{\text{mh}}\right)}{{\hat{h}}_{\min}}$$ (13)

The mean number of eigenvalues *M*_*h*_ obtained in each eigenvalue matrices was used as a feature since this value gives valuable information on signal shape (Li et al., 2021), helping to identify changes in the morphology. Finally, the mean and standard deviation of the *κ* were used as descriptors of the pixel distribution of the eigenvalues matrices.

##### 2.2.3.2 Energy-based features

Laws’ mask features are standard image processing based features used to measure the “Texture energy” of a group of pixels in an image. This method has been used in the past for feature extraction for biomedical images (Rachidi et al., 2008), speech recognition using spectrograms (Wang, 2014), texture classification (Dash and Jena, 2017), and as a method of segmentation based on the texture presented on the image (Kvyetnyy et al., 2017).

The principle of this method is to estimate the texture features using a set of texture energy transformations (Laws, 1980). This transformation detects the variation within a fixed-size window using different convolution masks that compute the energy of the image. This group of masks is invariant to changes in luminance, contrast, and rotation that allows the detection of textures under different conditions (Laws, 1980; Stockman and Shapiro, 2001).

All the convolution masks used for the energy texture estimation came from the following set of one-dimensional (1-D) kernels of five or three pixels:

• Kernels with length 3

$$L_{3}=\left[1,2,1\right],\;\;E_{3}=\left[1,0,-1\right],\;\;S_{3}=\left[1,-2,1\right]$$ (14)

• kernels with length 5

$$\begin{array}{ll} L_{5} & =\left[1,4,6,4,1\right],\;\;\\ E_{5} & =\left[-1,-2,0,2,1\right],\;\;\\ S_{5} & =\left[-1,0,2,0,-1\right],\\ W_{5} & =\left[-1,2,0,-2,1\right],\;\;\\ R_{5} & =\left[1,-4,6,-4,1\right] \end{array}$$

where L (Level) detects the average grey level, E (Edge) extracts edge features, S (Spot) extracts spots, W (Wave) extracts wave features, and R (Ripple) extracts ripples in the image (Laws, 1980). The convolution masks used for the feature extraction were generated by convoluting any vertical one-dimensional vector with a horizontal vector to generate the following 3 × 3 and 5 × 5 filters:

• 3 × 3 filters
$$\begin{array}{ccc} L_{3}^{T}L_{3} & E_{3}^{T}L_{3} & S_{3}^{T}L_{3}\\ L_{3}^{T}E_{3} & E_{3}^{T}E_{3} & S_{3}^{T}E_{3}\\ L_{3}^{T}S_{3} & E_{3}^{T}S_{3} & S_{3}^{T}S_{3} \end{array}$$

• 5 × 5 filters
$$\begin{array}{ccccc} L_{5}^{T}L_{5} & E_{5}^{T}L_{5} & S_{5}^{T}L_{5} & W_{5}^{T}L_{5} & R_{5}^{T}L_{5}\\ L_{5}^{T}E_{5} & E_{5}^{T}E_{5} & S_{5}^{T}E_{5} & W_{5}^{T}E_{5} & R_{5}^{T}E_{5}\\ L_{5}^{T}S_{5} & E_{5}^{T}S_{5} & S_{5}^{T}S_{5} & W_{5}^{T}S_{5} & R_{5}^{T}S_{5}\\ L_{5}^{T}W_{5} & E_{5}^{T}W_{5} & S_{5}^{T}W_{5} & W_{5}^{T}W_{5} & R_{5}^{T}W_{5}\\ L_{5}^{T}R_{5} & E_{5}^{T}R_{5} & S_{5}^{T}R_{5} & W_{5}^{T}R_{5} & R_{5}^{T}R_{5} \end{array}$$

The images obtained after convolution between each mask and the images should be normalized to make the descriptors contrast-independent. The normalization was made based on the implementation made by (Miroslav and Rodojevi´c, 2007), where all the images were normalized using the image min-max normalization as follows

$${\hat{I}}_{\mathrm{mask}}=\frac{I_{\mathrm{mask}}-\min\left(I_{\mathrm{mask}}\right)}{\max\left(I_{\mathrm{mask}}\right)-\min\left(I_{\mathrm{mask}}\right)}$$ (15)

After the normalization, each outputs *I*_*mask*_ were converted to a texture energy image (*E*_*mask*_) by using a moving non-linear window average of absolutes (Rachidi et al., 2008)

$$E_{\mathrm{mask}}\left(r,c\right)=\overset{c+7}{\underset{j=c-7}{\sum }}\overset{r+7}{\underset{i=r-7}{\sum }}|I_{\mathrm{mask}}\left(i,j\right)|$$ (16)

Finally, the mean (Eq. 17), standard deviation (Eq. 18), and entropy (Eq. 19) to each of the texture energy images obtained was computed to obtain a measurement of the global energy texture for each mask (Rachidi et al., 2008).

$$ME\left(E_{\mathrm{mask}}\right)=\frac{\sum_{i=0}^{M}\sum_{j=0}^{N}\left[E_{\mathrm{mask}}\left.\left(i,j\right)\right)\right]}{M\times N}$$ (17)

$$STD\left(E_{\mathrm{mask}}\right)=\sqrt{\frac{\sum_{i=0}^{M}\sum_{j=0}^{N}{\left(E_{\mathrm{mask}}\left(i,j\right)-Mean\right)}^{2}}{M\times N}}$$ (18)

$$EN\left(E_{\mathrm{mask}}\right)=\frac{\sum_{i=0}^{M}\sum_{j=0}^{N}{\left(E_{\mathrm{mask}}\left(i,j\right)\right)}^{2}}{M\times N}$$ (19)

##### 2.2.3.3 Central moment features

Statistical central moments are a set of features used to describe the spread and shape of the pixel’s distribution in an image (Grubbström and Tang, 2006) and are computed as

$${\hat{\mu }}_{k}=E{\left(I-\mu \right)}^{k}\;k=1,2,3,4$$ (20)

where *μ*_*k*_ represents the *k*th central moment about the mean *μ* of the spectrogram image *I*.

These features have been used for different applications where spectrograms are involved such as event classification and inference detection (Dennis et al., 2011; Mulimani and Koolagudi, 2018; Oh and Kim, 2019).

In this work, we extracted a set of features inspired by features used for audio event classification in (Mulimani and Koolagudi, 2018) derived from the second, third, and fourth central moment, described as follows:

$$\begin{array}{cc} F1 & =log_{10}\left(\sqrt{\mu_{2}}\right),\;\;\;\\ F2 & =\mu_{3},\;\;\;\\ F3 & =log_{10}\left(\mu_{4}\right),\;\;\;\\ F4 & =\sqrt{\hat{\mu_{2}}},\;\;\;\\ F5 & =\hat{\mu_{3}},\;\;\;\\ F6 & =\hat{\mu_{4}}, \end{array}$$ (21)

where $\sqrt{\mu_{2}}$ represents the standard derivation, *μ*_3_ is the skewness, and *μ*_4_ represents the kurtosis which indicates the flatness of the image histogram. The $\hat{\mu_{k}}$ with *k* = 1, 2, 3, 4 represent the central moments computed from the normalized spectrogram $\left(\hat{I}\right)$ .

$$\hat{I_{i,j}}=\frac{I\left(i,j\right)-\min\left(I\right)}{\max\left(I\right)-\min\left(I\right)}$$ (22)

### 2.3 Feature selection

The feature selection is a technique used to reduce the number of features by eliminating the irrelevant, redundant, and noisy features to improve the model performance (Kumar and Minz, 2014). In this study, we use the Maximum Relevance—Minimum Redundancy (MRMR) algorithm, which is a feature selection method that chooses $\ddot{S}$ number of features that has maximum relevance with respect to the target variable and minimum redundancy with respect to the features that have been selected at previous iterations (Zhao et al., 2019). We relied on the F-test correlation quotient (FCQ) variant of the Maximum Relevance—Minimum Redundancy (MRMR) algorithm to rank the features. This variant is based on the relevance of a feature to predict the desired variable, measured by the F-statistic between the feature and the target variable and the redundancy of the feature computed by the average Pearson correlation between the feature and all the other features.

$$FCQ_{\mathrm{score}}\left(f\right)=\frac{F\left(Y,f\right)}{\frac{1}{\ddot{S}}\sum_{s\in S}\rho \left(f,s\right)}$$ (23)

where *ρ*(*f*, *s*) is the Pearson correlation, *F*(*Y*, *f*) is the F-statistic and *Y* is the target variable to estimate, *S* the set of selected features, $\ddot{S}$ is the number of feature selected, *s* is a feature such as *s* ∈ *S* and *f* denotes a feature currently not selected (*f*∉*S*) (Zhao et al., 2019).

Finally, the number of features $\left(\ddot{S}\right)$ to be selected was chosen by a sensitivity analysis taking a different number of features which range between 1 and the total number of features to predict the cf-PWV using the proposed machine learning models with the default hyperparameters defined by the python library scikit-learn. Then, the set of features that produce the best mean *R* ^2^ performance of the models is selected to ensure high performance. Table 2 shows the number of features selected for each feature type, details on the feature selection can be found in the Supplementary Material.

**TABLE 2:** Number of features selected for BP and PPG spectrogram.

Signal	Location	SCSA	Energy	Statistical
BP	Radial	11	17	6
	Digital	11	15	6
	Brachial	14	5	5
PPG	Radial	11	25	6
	Digital	10	19	6
	Brachial	13	21	6

### 2.4 Machine learning models

To create the training and testing dataset for supervised machine learning models, the dataset was split into two different groups where the 70% of the total dataset was used for the training set, and the 30% left was used for the testing set. Finally, each of the three different features types were fed into the following machine learning methods: Random forest regression (RF), Gradient Boost Regressor (GBR), multilayer perceptron (MLP), Multiple Linear regression (MLR), and Suppor Vector Regression (SVR).

#### 2.4.1 Model training

A common practice in machine learning to increase the performance of the models is to standardize the features to have mean 0 and variance 1 using the z-score defined as follows,

$${\tilde{x}}_{i}^{\left(j\right)}=\frac{x_{i}^{\left(j\right)}-\mu_{i}}{\sigma_{i}}$$ (24)

where $x_{i}^{\left(j\right)}$ represents the value of the *i*th feature of the *j*th data point, *μ*_*i*_ represents the mean of each feature, and *σ*_*i*_ is the standard deviation of each feature. In addition, tuning the hyperparameters of the models helps to maximize the performance on the test data for given a specific problem (Elgeldawi et al., 2021). In this project, the hyperparameters optimization of the Machine learning models was made using a random search that has been used in the past for hyperparameter tuning (Jin et al., 2021; Garcia et al., 2022). This algorithm randomly selects different combinations of hyperparameters from a predefined space of values and tests the model’s performance model. Finally, the combination of hyperparameters with the best performance was selected. More information about the hyperparameters space and the values selected for each model can be found in the supplementary material. In combination with the hyperparameter tuning, a 5-fold cross-validation method was implemented to avoid over-fitting during the models’ training and hyperparameter tuning and increase the generalization capacity.

#### 2.4.2 Model evaluation

To evaluate the performance of the models, we used the R-squared (R^2^) value and the root mean square error (RMSE) between the actual value and the predicted by the model (Bahloul et al., 2021; Garcia et al., 2022).

$$R^{2}=1-\frac{\sum_{n=1}^{N}{\left(cf-PWV_{\mathrm{real}}^{n}-cf-PWV_{\mathrm{predicted}}^{n}\right)}^{2}}{\sum_{n=1}^{N}{\left(cf-PWV_{\mathrm{real}}^{n}-\mu \left(cf-PWV_{\mathrm{real}}\right)\right)}^{2}},$$ (25)

$$RMSE=\sqrt{\frac{\sum_{n=1}^{N}{\left(cf-PWV_{\mathrm{real}}^{n}-cf-PWV_{\mathrm{predicted}}^{n}\right)}^{2}}{N}},$$ (26)

where *μ* is a function that evaluates the mean of cf − PWV_real_ over N subjects.

### 2.5 Noise addition

To test the performance of the proposed methodology against noisy data, a high-frequency Gaussian white noise was added to the pulse wave signals to simulate the electrical noise found during the recording (Ban and Kwon, 2016). The typical cause for this type of noise is radio, TV, cellular, and distant lightning (Kularatna et al., 2019). The intensity of the noise was defined using the signal-to-noise ratio (SNR) defined as follows:

$$SNR=\frac{P_{s}}{P_{n}},$$ (27)

where *P*_*s*_ and *P*_*n*_ correspond to the power of the signal and *Gaussian* white noise, respectively (Bahloul et al., 2021; Garcia et al., 2022). The selected noise intensity for the BP signals is 20, 10, and 5 dB based on the values used by (Jin et al., 2021). For the PPG signals, we define the values as 65, 45, and 30 dB based on the values reported by (Maxim integrated, 2017) (Elsamnah et al., 2019).

## 3 Results

### 3.1 Noise-free case

This project proposed a novel methodology based on spectrogram representation of the signals to estimate the cf-PWV. Table 3 shows the result for the PPG spectrograms where the MLP and SVR models obtained the best results with a *R* ^2^ = 0.90 or higher and a *RMSE* = 0.71 or lower for the three features types, producing the best performance values of *R* ^2^ = 0.99 and *RMSE* = 0.16 for the SVR with energy features applied to the brachial location. In contrast, the MLR model shows the worst performance of *R* ^2^ = 0.73 and *RMSE* = 1.09 using statistical features extracted from the Brachial location. However, it is important to notice that in the case of the SCSA and energy features, the MLR models presented *R* ^2^ = 0.90 for higher and *RMSE* = 0.66 or lower, obtaining the best results of *R* ^2^ = 0.95 and *RMSE* = 0.47 for the energy features extracted from the Radial location. The feature type with the better overall performance in the estimation was the energy feature presenting a mean of *R* ^2^ = 0.97 and *RMSE* = 0.32 for all the models in the three different locations. Similarly, the result obtained for the BP spectrograms showed the best performance for the SVR and MLP models with at least a *R* ^2^ = 0.97 and a *RMSE* = 0.36 for all the features with a maximum difference of 0.02 for the *R* ^2^ and 0.19 between the features. On the other hand, the worst results were presented for the MLR models with a lower value of *R* ^2^ = 0.77 and *RMSE* = 1.00 for the Brachial location using the statistical features. As in the PPG spectrogram, the energy features presented the best overall result with a mean value of *R* ^2^ = 0.97 and *RMSE* = 0.36 for all the models in the three different locations. Finally, it is important to notice that all the features obtained a performance of *R* ^2^ between 0.90 and 0.99 for the BP and PPG spectrograms in the different locations.

**TABLE 3:** Free-noise results obtained for PPG and BP spectrogram.

Signal	Location	Feature	RF		GBR		MLP		MLR		SVR	
			RMSE	R2	RMSE	R2	RMSE	R2	RMSE	R2	RMSE	R2
PPG	Radial	Statistic	0.81	0.85	0.78	0.86	0.60	0.92	1.00	0.77	0.64	0.91
		SCSA	0.58	0.92	0.58	0.92	0.44	0.96	0.66	0.90	0.39	0.96
		Energy	0.39	0.97	0.30	0.98	0.25	0.99	0.47	0.95	0.25	0.99
	Digital	Statistic	0.83	0.84	0.82	0.84	0.61	0.91	0.99	0.77	0.65	0.90
		SCSA	0.59	0.92	0.59	0.92	0.44	0.96	0.57	0.93	0.37	0.97
		Energy	0.41	0.96	0.28	0.98	0.25	0.99	0.49	0.95	0.19	0.99
	Brachial	Statistic	0.82	0.84	0.82	0.84	0.64	0.90	1.09	0.73	0.69	0.89
		SCSA	0.54	0.93	0.54	0.93	0.41	0.96	0.57	0.93	0.35	0.97
		Energy	0.39	0.96	0.27	0.98	0.19	0.99	0.49	0.94	0.16	0.99
BP	Radial	Statistic	0.59	0.92	0.57	0.93	0.33	0.98	0.72	0.88	0.30	0.98
		SCSA	0.50	0.94	0.46	0.95	0.30	0.98	0.45	0.95	0.24	0.99
		Energy	0.37	0.97	0.25	0.99	0.22	0.99	0.55	0.93	0.17	0.99
	Digital	Statistic	0.58	0.92	0.55	0.93	0.32	0.98	0.76	0.87	0.30	0.98
		SCSA	0.51	0.94	0.48	0.95	0.33	0.97	0.46	0.95	0.26	0.98
		Energy	0.41	0.96	0.28	0.98	0.24	0.99	0.54	0.93	0.22	0.99
	Brachial	Statistic	0.74	0.87	0.71	0.88	0.32	0.98	1.00	0.77	0.32	0.98
		SCSA	0.54	0.93	0.54	0.93	0.28	0.98	0.51	0.94	0.25	0.99
		Energy	0.36	0.97	0.36	0.97	0.33	0.98	0.71	0.88	0.36	0.97

These results show the capacity of the spectrogram as signal representation for cf-PWV estimation using noise-free PPG and BP signals from the Radial, Brachial, and Digital locations. It is important to notice that BP shows a better performance with all three different features compared with the results obtained for the PPG where the energy-based features performed better than the other features for the three locations proposed. It is important to notice that the MLR model with SCSA and energy features obtained values of *R* ^2^ ≥ 0.90 showing a great capacity to obtain a linear relationship between the features extracted from the spectrogram and the cf-PWV. This is a great advantage since these linear models can allow an easier implementation of the model in real-life applications.

### 3.2 Noisy case

Table 4 shows the results obtained for the different levels of noise. The highest results obtained for each of the nose levels were *R* ^2^ = 0.98 and *RMSE* = 0.33 for the *SNR* = 65 using the MLP models with the energy features applied to the Brachial location. In addition, SCSA features applied in the brachial location presented the best result for the *SNR* = 45 with values *R* ^2^ = 0.96 and *RMSE* = 0.43 using the SVR model, and values of *R* ^2^ = 0.92 and *RMSE* = 0.60 for the *SNR* = 30 using the MLP and SVR models. In contrast, the worst results were obtained in all the cases by the MLP models using statistic features with values of *R* ^2^ = 0.76 and *RMSE* = 1.01 for the Radial location with *SNR* = 0.65, *R* ^2^ = 0.68 and *RMSE* = 1.17 for Digital and brachial location with *SNR* = 0.45, and *R* ^2^ = 0.65 and *RMSE* = 1.23 for Digital and brachial location with *SNR* = 0.30. For the BP spectrogram, The best values obtained were applied for Radial locations with values of *R* ^2^ = 0.98 and *RMSE* = 0.26 for the *SNR* = 20 using the GBR model with the energy features, *R* ^2^ = 0.98 and *RMSE* = 0.32 for the *SNR* = 10 using the SVR model with the SCSA features, and *R* ^2^ = 0.97 and *RMSE* = 0.38 for the *SNR* = 5 using the SVR model with the SCSA features. In contrast, similar to the PPG spectrograms, the lower results were obtained for the MLR models using the statistical features obtaining values of *R* ^2^ = 0.70 and *RMSE* = 1.14 for *SNR* = 20, *R* ^2^ = 0.68 and *RMSE* = 1.18 for *SNR* = 10, and *R* ^2^ = 0.69 and *RMSE* = 1.16 for *SNR* = 5.

**TABLE 4:** Results obtained for noisy data.

Signal	Location	Feature	Noise level	RF		GBR		MLP		MLP		SVR	
				RMSE	R2	RMSE	R2	RMSE	R2	RMSE	R2	RMSE	R2
PPG	Radial	Statistic	SNR = 65	0.81	0.85	0.80	0.85	0.61	0.91	1.01	0.76	0.65	0.90
			SNR = 45	0.90	0.81	0.91	0.81	0.80	0.85	1.13	0.70	0.82	0.85
			SNR = 30	0.93	0.80	0.97	0.79	0.95	0.79	1.14	0.70	0.93	0.80
		SCSA	SNR = 65	0.60	0.92	0.58	0.92	0.43	0.96	0.66	0.90	0.40	0.96
			SNR = 45	0.64	0.91	0.61	0.91	0.48	0.95	0.69	0.89	0.46	0.95
			SNR = 30	0.72	0.88	0.71	0.88	0.61	0.92	0.80	0.86	0.64	0.91
		Energy	SNR = 65	0.43	0.96	0.40	0.96	0.40	0.96	0.47	0.95	0.48	0.95
			SNR = 45	0.58	0.92	0.57	0.93	0.63	0.91	0.77	0.86	0.77	0.87
			SNR = 30	0.68	0.89	0.67	0.90	0.76	0.87	1.00	0.77	0.90	0.82
	Digital	Statistic	SNR = 65	0.82	0.85	0.82	0.85	0.62	0.91	0.99	0.77	0.65	0.90
			SNR = 45	0.87	0.83	0.88	0.82	0.76	0.87	1.17	0.68	0.78	0.86
			SNR = 30	0.90	0.81	0.90	0.81	0.88	0.82	1.23	0.65	0.88	0.82
		SCSA	SNR = 65	0.59	0.92	0.60	0.92	0.45	0.95	0.58	0.92	0.37	0.97
			SNR = 45	0.60	0.92	0.64	0.91	0.50	0.94	0.68	0.89	0.45	0.95
			SNR = 30	0.78	0.86	0.76	0.87	0.63	0.91	0.78	0.86	0.66	0.90
		Energy	SNR = 65	0.46	0.95	0.40	0.96	0.34	0.97	0.41	0.96	0.46	0.95
			SNR = 45	0.62	0.91	0.57	0.92	0.66	0.90	0.69	0.89	0.77	0.87
			SNR = 30	0.72	0.88	0.69	0.89	0.77	0.86	0.90	0.82	0.96	0.80
	Brachial	Statistic	SNR = 65	0.79	0.86	0.78	0.86	0.59	0.92	0.99	0.78	0.62	0.91
			SNR = 45	0.87	0.83	0.88	0.82	0.76	0.87	1.17	0.68	0.78	0.86
			SNR = 30	0.90	0.81	0.90	0.81	0.88	0.82	1.23	0.65	0.88	0.82
		SCSA	SNR = 65	0.55	0.93	0.55	0.93	0.39	0.96	0.57	0.92	0.35	0.97
			SNR = 45	0.57	0.93	0.56	0.93	0.45	0.95	0.62	0.91	0.43	0.96
			SNR = 30	0.73	0.88	0.72	0.88	0.60	0.92	0.74	0.87	0.60	0.92
		Energy	SNR = 65	0.39	0.97	0.35	0.97	0.33	0.98	0.40	0.96	0.42	0.96
			SNR = 45	0.55	0.93	0.53	0.93	0.63	0.91	0.68	0.89	0.71	0.89
			SNR = 30	0.72	0.88	0.69	0.89	0.77	0.86	0.90	0.82	0.96	0.80
BP	Radial	Statistic	SNR = 20	0.62	0.91	0.62	0.91	0.39	0.96	0.76	0.87	0.38	0.97
			SNR = 10	0.68	0.89	0.69	0.89	0.55	0.93	0.89	0.82	0.52	0.94
			SNR = 5	0.71	0.88	0.70	0.89	0.60	0.92	0.92	0.81	0.61	0.91
		SCSA	SNR = 20	0.52	0.94	0.49	0.94	0.30	0.98	0.46	0.95	0.27	0.98
			SNR = 10	0.55	0.93	0.51	0.94	0.37	0.97	0.51	0.94	0.32	0.98
			SNR = 5	0.58	0.92	0.55	0.93	0.39	0.96	0.55	0.93	0.38	0.97
		Energy	SNR = 20	0.31	0.98	0.26	0.98	0.28	0.98	0.29	0.98	0.33	0.98
			SNR = 10	0.43	0.96	0.37	0.97	0.42	0.96	0.47	0.95	0.47	0.95
			SNR = 5	0.50	0.94	0.45	0.95	0.50	0.94	0.57	0.93	0.56	0.93
	Digital	Statistic	SNR = 20	0.62	0.91	0.61	0.92	0.39	0.97	0.80	0.85	0.38	0.97
			SNR = 10	0.66	0.90	0.66	0.90	0.54	0.93	0.90	0.81	0.55	0.93
			SNR = 5	0.69	0.89	0.68	0.89	0.64	0.91	0.92	0.81	0.63	0.91
		SCSA	SNR = 20	0.53	0.93	0.51	0.94	0.34	0.97	0.47	0.95	0.29	0.98
			SNR = 10	0.56	0.93	0.54	0.93	0.36	0.97	0.54	0.93	0.34	0.97
			SNR = 5	0.60	0.92	0.57	0.92	0.41	0.96	0.58	0.92	0.39	0.97
		Energy	SNR = 20	0.34	0.97	0.28	0.98	0.27	0.98	0.31	0.98	0.34	0.97
			SNR = 10	0.48	0.95	0.39	0.97	0.41	0.96	0.47	0.95	0.48	0.95
			SNR = 5	0.53	0.94	0.46	0.95	0.50	0.94	0.56	0.93	0.56	0.93
	Brachial	Statistic	SNR = 20	0.79	0.86	0.79	0.86	0.46	0.95	1.14	0.70	0.46	0.95
			SNR = 10	0.79	0.86	0.77	0.86	0.61	0.92	1.18	0.68	0.64	0.91
			SNR = 5	0.80	0.85	0.79	0.85	0.65	0.90	1.16	0.69	0.71	0.88
		SCSA	SNR = 20	0.64	0.91	0.57	0.93	0.31	0.98	0.57	0.93	0.29	0.98
			SNR = 10	0.67	0.90	0.61	0.91	0.36	0.97	0.66	0.90	0.37	0.97
			SNR = 5	0.71	0.89	0.64	0.90	0.39	0.96	0.75	0.87	0.44	0.95
		Energy	SNR = 20	0.47	0.95	0.35	0.97	0.33	0.98	0.44	0.96	0.45	0.96
			SNR = 10	0.57	0.93	0.46	0.95	0.45	0.95	0.58	0.92	0.54	0.93
			SNR = 5	0.62	0.91	0.51	0.94	0.57	0.93	0.69	0.89	0.63	0.91

These results show that even with different levels of white gaussian noise added to the original signals, the proposed methodology obtained *R* ^2^ ≥ 0.90 for the PPG signals with the different levels of noise, using the MLP and SVR models for the different locations. In contrast, similar to the case of noise-free, the BP signals obtained better results, presenting an *R* ^2^ ≥ 0.90 using the MLP model for the three different features in the proposed locations. Furthermore, in the case of BP signals, the MLR model obtained accurate results for the cf-PWV.

## 4 Discussion

This study investigated a novel methodology to estimate the cf-PWV based on the application of the spectrogram representation of single PPG or BP signals extracted from a peripheral location. The use of the spectrogram representation for the analysis of biomedical signals such as PPG had been studied before as input for data-driven approaches like the classification of peripheral diseases by (Allen et al., 2021), or biometric recognition, (Donida Labati et al., 2021). For this reason, in this project, the use of the spectrogram from BP or PPG signals to estimate the cf-PWV values is investigated as a novel methodology to take advantage of the frequency and temporal information encoded in the spectrogram matrix.

In this project, three feature types based on the Schrodinger spectrum, the image’s energy texture, and the image’s statistical distribution were combined with different machine learning algorithms to estimate the carotid-to-femoral pulse wave velocity (cf-PWV). The results for the noisy-free signals presented a mean value of *R* ^2^ = 0.92 and *RMSE* = 0.54 for the PPG spectrograms and *R* ^2^ = 0.95 and *RMSE* = 0.44 for the BP spectrograms for the noisy-free signals extracted from the Radial, Digital and Brachial location, showing the spectrogram’s capacity to encode valuable information that can be extracted to estimate the cf-PWV presenting. The energy-based features using Law’s masks presented the best performance for the PPG and BP signals with values of *R* ^2^ = 0.99 and *RMSE* = 0.16 for the SVR applied to PPG spectrogram the Brachial location and *R* ^2^ = 0.99 and *RMSE* = 0.17 for the SVR applied to BP spectrogram the Brachial location. Nevertheless, it is important to recall that all three different feature types obtained one or more models with at least a *R* ^2^ = 0.90 and *RMSE* = 0.64 for all the locations. These results show the feasibility of the different types of features to extract valuable information from a spectrogram created using noisy-free signals to estimate the cf-PWV values.

In contrast, for the noisy cases, the PPG spectrograms presented values of *R* ^2^ = 0.92 and *RMSE* = 0.56 for the *SNR* = 65, *R* ^2^ = 0.88 and *RMSE* = 0.70 for the *SNR* = 45, and *R* ^2^ = 0.82 and *RMSE* = 0.84 for the *SNR* = 30, showing a decrease in the performance for the *SNR* = 45 and *SNR* = 30 cases of 0.04 and 0.10 for the *R* ^2^, and 0.16 and 0.30 for the *RMSE*. It is important to notice that the SCSA features presented the best overall performance for the noisy cases, obtaining the best mean results value of *R* ^2^ = 0.94 and *RMSE* = 0.50 for MLP and SVR models applied for the different noisy PPG spectrograms and *R* ^2^ = 0.97 and *RMSE* = 0.34 for the SVR model applied for the noisy BP spectrograms, obtaining in a value of *R* ^2^ = 0.90 or higher for each of the noise level presented in this study. However, it is important to notice that the SCSA method presented a high computational complexity to compute the features for each combination of signals (PPG or BP) and location (Radial, Digital, Brachial). For this reason, it is essential to develop future works to reduce this complexity to extend this method for real applications where time and computational cost play an essential role.

Previous studies have been using machine learning or deep learning models to estimate the cf-PWV based on PPG or BP signals (Tavallali et al., 2015; Jin et al., 2021; Li et al., 2022). However, a direct comparison between our work and many of the previous studies cannot be made given that these studies use real data for the estimation, in contrast with the *in silico* data used in this study. Nevertheless, the study made by (Jin et al., 2021) used the same Blood Pressure *in silico* signals from the Radial location for the noisy case. In this study, the authors proposed the use of an LSTM deep-learning model to estimate the cf-PWV, obtaining an *R* ^2^ ≥ 0.98 and a *RMSE* ≤ 0.24. In contrast, the proposed method obtained a similar performance of an *R* ^2^ ≥ 0.97 and a *RMSE* ≤ 0.38.

It is important to notice that the model parameters used for the generation of the *in silico* pulse wave signals were changed with age, allowing the investigation of the effects of aging in the estimation of cf-PWV. Previous studies had demonstrated that there could exist a decrease in the performance of the estimation for high PWV values associated with the sensitivity to variations in the transit time during the cf-PWV estimation (Li et al., 2022; Jin et al., 2021). This same behavior was noticed in this project for some of the models where there is an increase of the error estimation for higher values of cf-PWV (usually higher than 9 $\frac{m}{s}$ ), these values are presented for virtual patients between 55 and 75 years old (Charlton et al., 2019). h Nevertheless, the models with the best performances (*R* ^2^ ≥ 0.98) do not present this increase of error for the high cf-PWV, showing a great capacity to estimate the cf-PWV for all the different ages (25–75) without presenting an important increase in the error produced by the age of the virtual patient. These results are similar to the results reported in Jin et al. (2021) where they use the LSTM for the noisy data estimation obtaining a great capacity to estimate the cf-PWV regarding the age of the virtual patient.

Even if these results are promising, it is crucial to consider the different limitations presented in this project. The principal limitation is the use of *in silico* data rather than real data collected from a specific human population. Nevertheless, the *in silico* data allows us to achieve an initial validation of the proposed methodology, whose results will permit us to proceed with the use of real data. Another limitation of this project is the spectrogram representation made using one-cycle signals; given that the real PPG and BP signals present multiple cycles, this will change the spectrogram image obtained, and this could make the features computed in this work may not work for the multi-cycle representation. However, future work will be done toward solving this limitation to validate the proposed methodology to obtain a more realistic analysis of the feasibility of the spectrogram to estimate the cf-PWV.

## 5 Conclusion

This paper investigates a new methodology to estimate cf-PWV based on the spectrogram representation obtained from BP or PPG signals taken from peripheral signals using machine learning models. The proposed approach incorporates three different types of features to probe the feasibility of the spectrogram to accurately estimate the cf-PWV. The results prove that the three different methods could obtain good performance, where the energy features showed the best performance for all the models without noise and the SCSA presented the best results against the noise levels proposed in the study. In the future, further validation of the proposed methodology in real human signals needs to be conducted to overcome the limitation of using *in silico* data with one cardiac cycle. This proposed method may be implemented for personal healthcare applications upon successful clinical validation. Also, it can open the door for future investigations of new machine learning methods and feature extraction techniques to improve the estimation of cf-PWV based on spectrogram representation. The code is available at https://github.com/EMANG-KAUST/Spectrogram_AS_Frontiers. We welcome developments to the existing code or contributions of new algorithms for inclusion in future versions of the arterial stiffness prediction platform.

## Data Availability

The original contributions presented in the study are included in the article/Supplementary Material, further inquiries can be directed to the corresponding author.

## Footnotes

1
https://peterhcharlton.github.io/pwdb/index.html
