# Supplementary material for: A learning-based image processing approach for pulse wave velocity estimation using spectrogram from peripheral pulse wave signals: An in silico study
Source: Front Physiol. 2023 Mar 3;14:1100570. doi: 10.3389/fphys.2023.1100570 (PMC10020726; doi:10.3389/fphys.2023.1100570)
Supplement: Supplementary file 1 [file DataSheet1.pdf]

Supplementary Material

1 SUPPLEMENTARY TABLES

1.1 Spectrogram's parameters selection

Table S1. Results obtained for the spectrogram quality metrics.

Window	Overlapping	Size	Qt	Qf	Qtf
Hamming	0	20	0.41	1.30	0.53
		50	0.66	1.22	0.80
		100	1.30	1.21	1.57
		166	8.76	1.22	10.68
		250	1.54e+13	1.22	1.87e+13
	60	20	0.33	1.32	0.43
		50	0.48	1.25	0.60
		100	0.78	1.23	0.96
		166	1.30	1.22	1.58
		250	8.78	1.22	10.72
	95	20	0.30	1.41	0.43
		50	0.42	1.28	0.54
		100	0.65	1.24	0.81
		166	1.03	1.23	1.27
		250	1.73	1.22	2.12
Kaiser	0 ($\alpha = 0.5$)	20	0.54	1.30	0.70
		50	0.77	1.22	0.94
		100	1.09	1.22	1.33
		166	1.49	1.22	1.83
		250	5.29e+12	1.22	6.43e+12
	61 ($\alpha = 3$)	20	0.32	1.32	0.42
		50	0.46	1.27	0.59
		100	0.76	1.24	0.94
		166	1.30	1.22	1.58
		250	19.13	1.22	23.35
	75 ($\alpha = 5$)	20	0.31	1.26	0.39
		50	0.45	1.26	0.56
		100	0.69	1.24	0.86
		166	1.30	1.22	1.58
		250	19.13	1.22	23.35

1.2 SCSA parameter selection

Table S2. SCSA parameters for PPG spectrograms.

Signal	Location	Noise-free		SNR=65		SNR=45		SNR=30	
		h	γ	h	γ	h	γ	h	γ
PPG	Brachial	0.1259	4	0.1259	4	0.1258	4	0.2000	2
	Radial	0.1260	4	0.1260	4	0.1259	4	0.2000	2
	Digital	0.1258	4	0.1258	4	0.1256	4	0.2000	4

Table S3. SCSA parameters for BP spectrograms.

Signal	Location	Noise-free		SNR=20		SNR=10		SNR=5	
		h	γ	h	γ	h	γ	h	γ
BP	Brachial	0.1264	4	0.1263	4	0.1262	4	0.1255	4
	Radial	0.1266	4	0.1265	4	0.1264	4	0.1263	2
	Digital	0.1266	4	0.1264	4	0.126	3	0.1264	4

1.3 Hyperparameter spaces

Table S4. Values used for hyperparameter tuning

Models	Parameters	Values
Random Forest (RF)	Number of estimators	min:50 , max:200
	Number of features to consider at every split	auto, sqrt , log2 ,1,2,5
	Minimum number of samples required to split a node	2,5, 10,15,20
	Minimum number of samples required at each leaf node	2,5, 10,15,20
	Apply bootstrap	True, False
Gradient Boost Regression (GB)	Loss function	ls, lad, huber
	Learning rate	0.01, 0.02, 0.05, 0.1
	Number of trees in random forest	min:100 , max:1000
	Number of features to consider at every split	auto, sqrt
	Maximum number of levels in tree	min:2 , max:10
	Minimum number of samples required to split a node	40, 60, 80
	Minimum number of samples required at each leaf node	20, 30, 40
Multiple Layer Perceptron (MLP)	Hidden layer sizes	(10,30,10), (20,), (15,11)
	Activation function	tanh, relu
	Solver	sgd, adam, lbfgs
	Alpha	0.0001, 0.001 ,0.01,0.1
	Learning rate	constant, adaptive
Multiple Linear Regression (MLR)	Fit intercept	True, False
Support Vector Regressor (SVR)	C parameters	min:100 , max:400
	Kernel	linear, rbf, sigmoid

1.4 Feature selection

The feature selection was made by using the feature ranking method FQC and then a sensitivity analysis based on the mean R^2 value.

1.4.1 SCSA features selection

Tables S5 and S6 shown the top 5 SCSA features from PPG and BP spectrogram respectively. As is shown, the matrix with more features for the BP and PPG spectrogram was the sum matrix that combines information from the row eigenvalues and the columns eigenvalues giving more information about the general spectrum of the image. In addition, the features based on the ratio between h and the κ (R and MR) were the most repeated features in the top 5 showing relevance of the relation between the h parameters and the eigenvalues to predict the cf-PWV.

Table S5. Top 5 SCSA features ranked on PPG spectrograms

Singal	Feature ranking				
Radial	$INV3_{sum}$	MR_{sum}	R_{row}	$E2_{sum}$	$E2_{row}$
Digital	$INV2_{sum}$	R_{row}	MR_{sum}	$E1_{row}$	$INV3_{sum}$
Brachial	$Mean(\kappa)_{sum}$	R_{row}	MR_{column}	$INV3_{sum}$	$K1_{row}$

Table S6. Top 5 SCSA features ranked on BP spectrograms

Singal	Feature ranking				
Radial	$STD(\kappa)_{column}$	MR_{row}	MR_{sum}	$INV3_{sum}$	MR_{column}
Digital	$E2_{column}$	MR_{row}	MR_{sum}	$INV3_{sum}$	MR_{column}
Brachial	$INV1_{row}$	$E2_{row}$	MR_{column}	$INV3_{sum}$	MR_{sum}

The best feature for the PPG spectrogram were $INV3_{sum}$, $INV2_{sum}$, and $Mean(\kappa)_{sum}$ for the Radial, Digital and Brachial locations respectively. Additionally, the best features for the BP spectrogram were the $STD(\kappa)_{column}$, $E2_{column}$, and $INV1_{row}$ for the Radial, Digital and Brachial locations respectively. This results shows the importance of the different invariants (INV) to describe changes in the spectrograms related to estimation of the cf-PWV values.

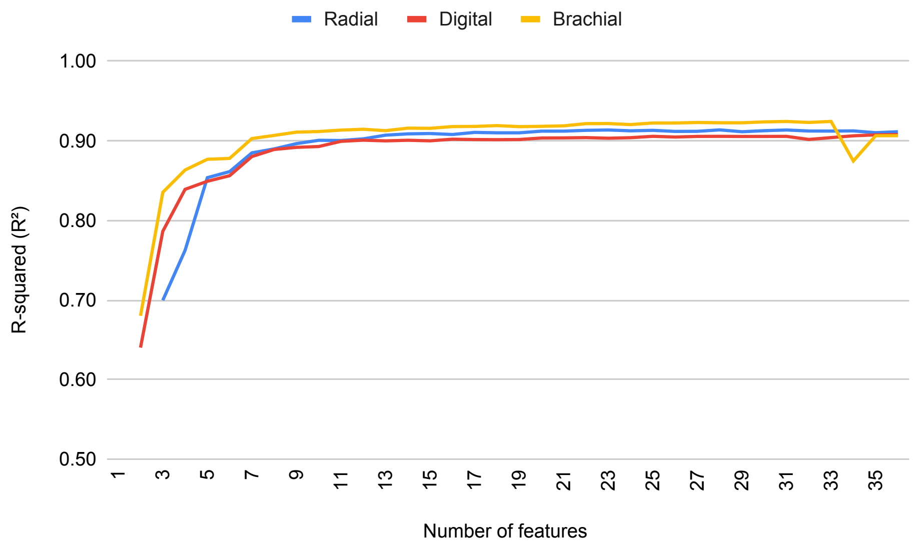

Figure S1. Sensitivity analysis for the PPG spectrogram using SCSA-based features

After obtain the feature ranking, a sensitivity analysis was made to select the number of features to use (Figure S1 and S2). Figure S1 show the result for PPG spectrograms were the best R^2 values obtained were 0.91 for the best 11 features for the Radial location, and the best 26 for the Digital location, and 0.92 for the best 13 features. For the Radial and Brachial features the number of features selected were the values named before were the R^2 obtain the maximum value and represent less than 40% of the original feature space. However, for the Digital spectrogram the best 10 features were selected given that the value obtained

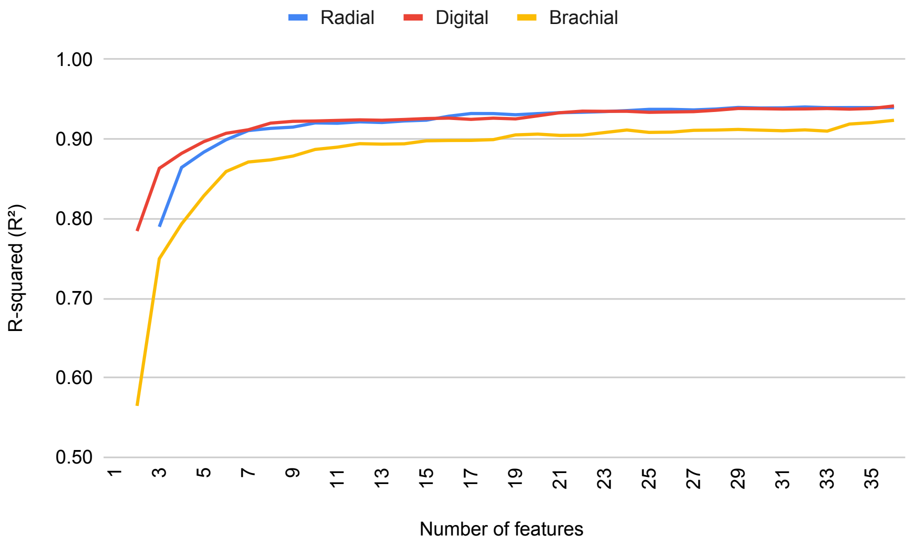

Figure S2. Sensitivity analysis for the BP spectrogram using SCSA-based features

(0.90) was just only 0.01 less than the maximum and using at least 16 features less. This selection helped to reduce the model complexity and reduce the computational complexity caused by SCSA.

Finally, it can be see in figure S2 that Radial and Digital BP spectrograms presented a similar behavior where the maximum value of R^2 was 0.94. For both signals the number of features selected was 11 with a R^2 of 0.92 using at least 15 and 20 features less and only having a R^2 lower by 0.02. Additionally, the best 14 features with a R^2 of 0.90 were selected from Brachial given that the maximum value of 0.92 (just 0.02 more) was obtained by using 33 features. As it was mentioned in the PPG feature selection, reduce the number of features reduces the computational cost of this method making this method more suitable to be use in the future in more applications where the computational cost can be a limitation. Table S7 show the number of SCSA selected.

Table S7. Number of SCSA features selected for BP and PPG spectrogram

Signal	Location	No.feature
	Radial	11
BP	Digital	11
	Brachial	14
	Radial	11
PPG	Digital	10
	Brachial	13

1.4.2 Energy features selection

Table S8 shows the top 5 ranked features from the 102 computed to each PPG spectrogram. It can be notice that only Radial PPG spectrogram have features extracted from the 3x3 mask being the standard derivation form the E_3L_3 mask the best feature obtained. Contrary, Digital and Brachial signal doesn't had any 3x3 mask between the top five obtaining as the best the standard deviation for the L_5W_5 and W_5L_5 5x5 masks respectively. It is important notice that the kernel W used to extract features of waves, is the most repeated kernel in the top five ranking for the three different locations. This indicate that the wave patterns are relevant in the prediction of the cf-PWV.

Table S8. Top 5 Envergy features ranked on PPG spectrograms

Singal	Top 5 feature ranking				
Radial	STD_{E3L3}	EN_{R5W5}	ME_{S5W5}	EN_{S3E3}	ME_{W5R5}
Digital	STD_{L5W5}	ME_{W5L5}	ME_{W5R5}	STD_{L5R5}	ME_{L5W5}
Brachial	STD_{W5L5}	ME_{L5S5}	ME_{W5R5}	STD_{L5R5}	EN_{E5R5}

Table S9. Top 5 Enenrgy features ranked on BP spectrograms

Singal	Top 5 feature ranking				
Radial	EN_{S5L5}	STD_{E5S5}	ME_{S5R5}	EN_{L5R5}	ME_{L5R5}
Digital	EN_{S5L5}	STD_{R5S5}	STD_{R5R5}	ME_{E3S3}	ME_{S3E3}
Brachial	STD_{L5W5}	ME_{E3E3}	ME_{R5R5}	STD_{S5L5}	STD_{L5S5}

For the BP signals, it can be seen in table S9 that the entropy from the image with the filter S_5L_5 obtained the best ranking for the Radial and Digital location, and for the Brachial location the standard deviation from the image with the filter L_5W_5. It can be seen that the kernel S used to extract features of spots is the most repeated kernel in the top five features for the locations studied.

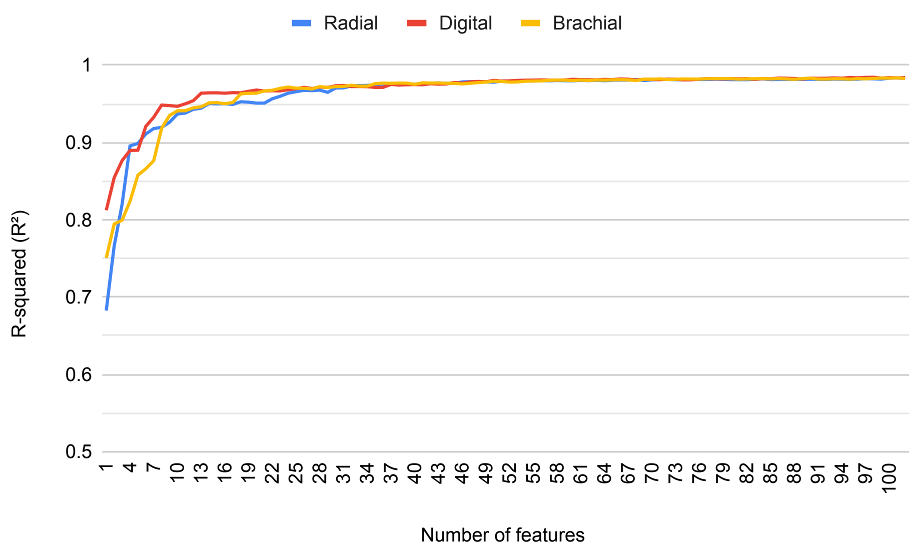

Figure S3. Sensitivity analysis for the PPG spectrogram using energy-based features

Figure S3 shows the sensitivity analysis made to select the number of feature to use for the PPG spectrogram. It can been observed that the three different location obtain a similar behavior where the highest R^2 was 0.98 using 36 features for Radial, 37 features for Digital, and 35 for Brachial. However, in order to reduce the possibility to overfitting, 25, 19, and 21 features were selected to each location respectively using 11 , 18, and 14 feature less and only obtaining a R^2 of 0.01 less than the maximum.

Based on figure S4 the number of features selected for the Brachial BP spectrogram was 5 obtaining a R^2 of 0.95. This decision was made given that the higher R^2 of 0.97 found for 46 to 102 features was only 0.02 higher but using at least 41 more features increasing the possibility to present overfitting in the models. In the same way, the best 17 and 15 features were selected for the Radial and Digital locations

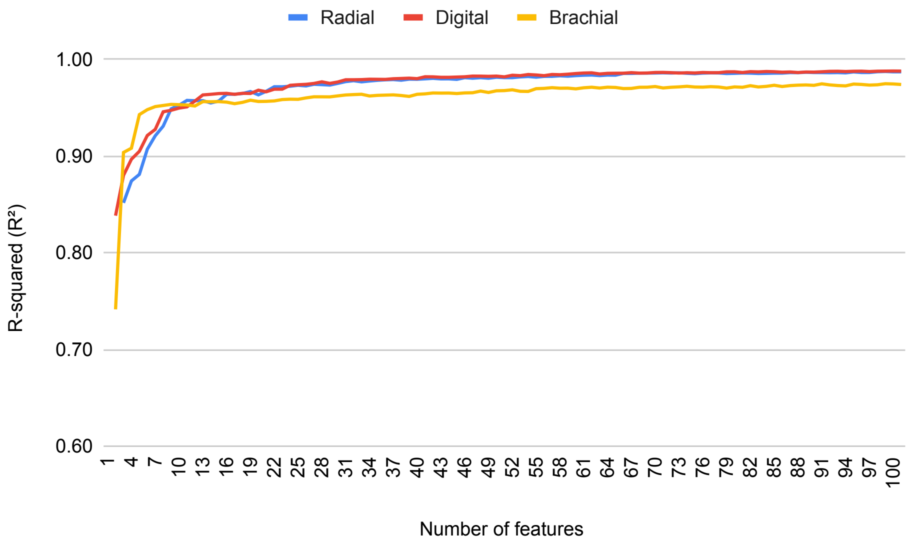

Figure S4. Sensitivity analysis for the BP spectrogram using energy-based features

respectively obtaining a R^2 of 0.97. This correspond to use at least 47 and 44 less features that the 64 and 59 features uses to obtain a R^2 of 0.99 respectively. Table S10 shows the number of features selected for the BP and PPG spectrograms.

Table S10. Number of Energy-based features selected for BP and PPG spectrogram

Signal	Location	No.feature
	Radial	17
BP	Digital	15
	Brachial	5
	Radial	25
PPG	Digital	19
	Brachial	21

1.4.3 Statistic feature selection

Table S11 shows the raking obtained for the six statistical features computed for the PPG spectrogram. As is shown, the first feature (F1) corresponding to the logarithmic of the standard derivation of the spectrogram obtained the best ranking for the Radial and Digital locations being the most relevant feature in both cases. This feature also obtained the third-best place for the Brachial spectrogram being the only feature between the top three for the three locations proposed in this study. On the other hand, the fourth feature (F4) related to the standard derivation of the normalized spectrogram was the only feature that never ranked between the top three.

Table S12 shows the feature raking obtained for the BP spectrogram. In contrast with the result obtained for the PPG signals, the F4 feature has relevance for the three locations being the first ranked for the Radial and the Brachial. Furthermore, table S12 shows that the BP spectrogram obtained similar ranking features for all the locations; where F3, F5, and F6 were ranked as fourth, fifth, and sixth for the three locations, and the features F1, F2, and F6 were in the top three in all the locations. It is important to notice that the Radial and Digital spectrograms obtained the same ranking for the features.

Table S11. Feature ranking for Statistic features on PPG spectrograms

Singal	Feature ranking					
Radial	F1	F2	F5	F3	F4	F6
Digital	F1	F3	F5	F4	F6	F2
Brachial	F6	F3	F1	F4	F5	F2

Table S12. Feature ranking for Statistic features on BP spectrograms

Singal	Feature ranking					
Radial	F4	F2	F1	F3	F5	F6
Digital	F4	F2	F1	F3	F5	F6
Brachial	F1	F2	F4	F3	F5	F6

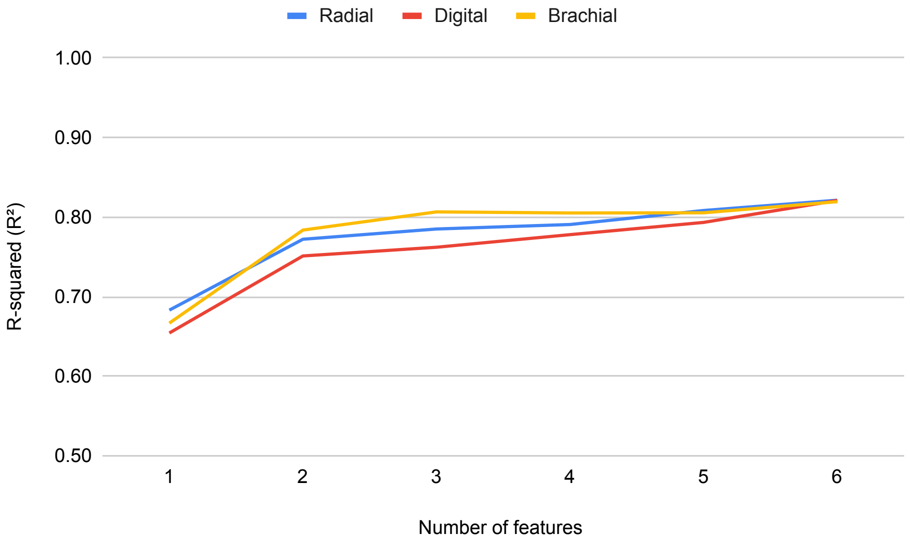

Figure S5. Sensitivity analysis for the PPG spectrogram using statistical-based features

Finally, figure S5 shows the mean R^2 obtained from all the ML algorithms using different numbers of features. As can be seen, for the three locations, the best results it was obtained using the 6 best features with a R^2 of 0.82. The number of 6 features was selected given that there is an improvement compared to the other results, even is this improvement is only by 0.01 compared with using 5 features. In contrast to the selection made for the Energy-based features, in this case it was decided gave more relevance to the R^2 value over the number of features because the number of features used was still low compared to other methods, for this reason there are low possibility to produce overfitting caused by the model complexity even if we used all the features.

On the other side, figure S6 shows the results of the sensitivity analysis made for the BP spectrogram. The Radial and Digital spectrogram presented a similar behavior than the PPG were the performance of the model increases with an increase in the feature used until reached the best value of 0.90 . For this reason the maximum number of features (6) were selected for this locations. However, for the Brachial BP the best 5 features were selected given that the maximum value of 0.86 were reached with this features, and when

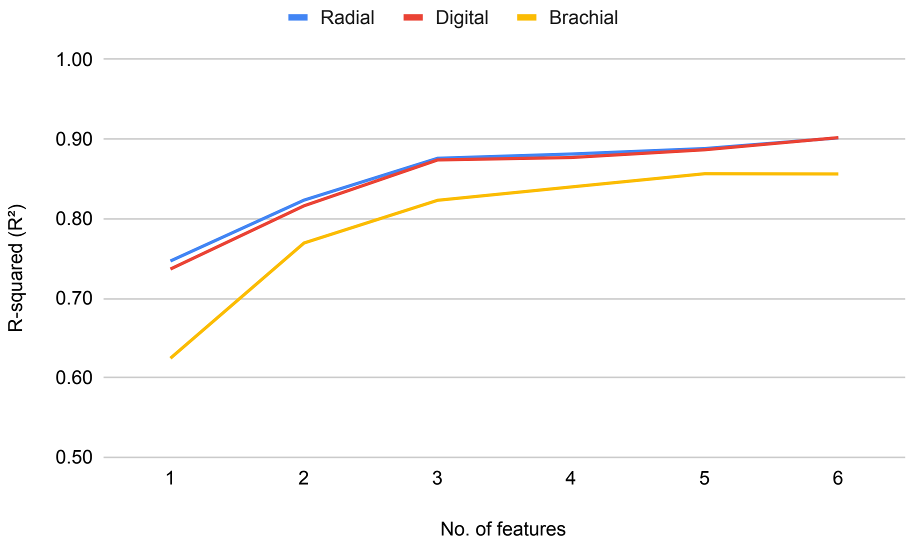

Figure S6. Sensitivity analysis for the PPG spectrogram using statistical-based features

Table S13. Number of Energy-based features selected for BP and PPG spectrogram

Signal	Location	No.feature
	Radial	6
BP	Digital	6
	Brachial	5
	Radial	6
PPG	Digital	6
	Brachial	6

the 6 best feature was added the models performance don't improved meaning that this feature don't have impact in the final result. Table S13 shows the number of Statistical features selected for each location.
